# A new high-performance liquid chromatography-tandem mass spectrometry method for the determination of paclitaxel and 6α-hydroxy-paclitaxel in human plasma: Development, validation and application in a clinical pharmacokinetic study

**DOI:** 10.1371/journal.pone.0193500

**Published:** 2018-02-23

**Authors:** Bianca Posocco, Mauro Buzzo, Andrea Follegot, Luciana Giodini, Roberto Sorio, Elena Marangon, Giuseppe Toffoli

**Affiliations:** 1 Experimental and Clinical Pharmacology, CRO- National Cancer Institute, Aviano, Pordenone, Italy; 2 Oncology Unit B, CRO- National Cancer Institute, Aviano, Pordenone, Italy; Pacific Northwest National Laboratory, UNITED STATES

## Abstract

Paclitaxel belongs to the taxanes family and it is used, alone or in multidrug regimens, for the therapy of several solid tumours, such as breast-, lung-, head and neck-, and ovarian cancer. Standard dosing of chemotherapy does not take into account the many inter-patient differences that make drug exposure highly variable, thus leading to the insurgence of severe toxicity. This is particularly true for paclitaxel considering that a relationship between haematological toxicity and plasma exposure was found. Therefore, in order to treat patients with the correct dose of paclitaxel, improving the overall benefit–risk ratio, Therapeutic Drug Monitoring is necessary. In order to quantify paclitaxel and its main metabolite, 6α-hydroxy-paclitaxel, in patients’ plasma, we developed a new, sensitive and specific HPLC–MS/MS method applicable to all paclitaxel dosages used in clinical routine. The developed method used a small volume of plasma sample and is based on quick protein precipitation. The chromatographic separation of the analytes was achieved with a SunFire^™^ C18 column (3.5 μM, 92 Å, 2,1 x 150 mm); the mobile phases were 0.1% formic acid/bidistilled water and 0.1% formic acid/acetonitrile. The electrospray ionization source worked in positive ion mode and the mass spectrometer operated in selected reaction monitoring mode. Our bioanalytical method was successfully validated according to the FDA-EMA guidelines on bioanalytical method validation. The calibration curves resulted linear (R^2^ ≥0.9948) over the concentration ranges (1–10000 ng/mL for paclitaxel and 1–1000 ng/mL for 6α-hydroxy-paclitaxel) and were characterized by a good accuracy and precision. The intra- and inter-day precision and accuracy were determined on three quality control concentrations for paclitaxel and 6α-hydroxy-paclitaxel and resulted respectively <9.9% and within 91.1–114.8%. In addition, to further verify the assay reproducibility, we tested this method by re-analysing the incurred samples. This bioanalytical method was employed with success to a genotype-guided phase Ib study of weekly paclitaxel in ovarian cancer patients treated with a wide range of drug’s dosages.

## Introduction

**Fig 1:**
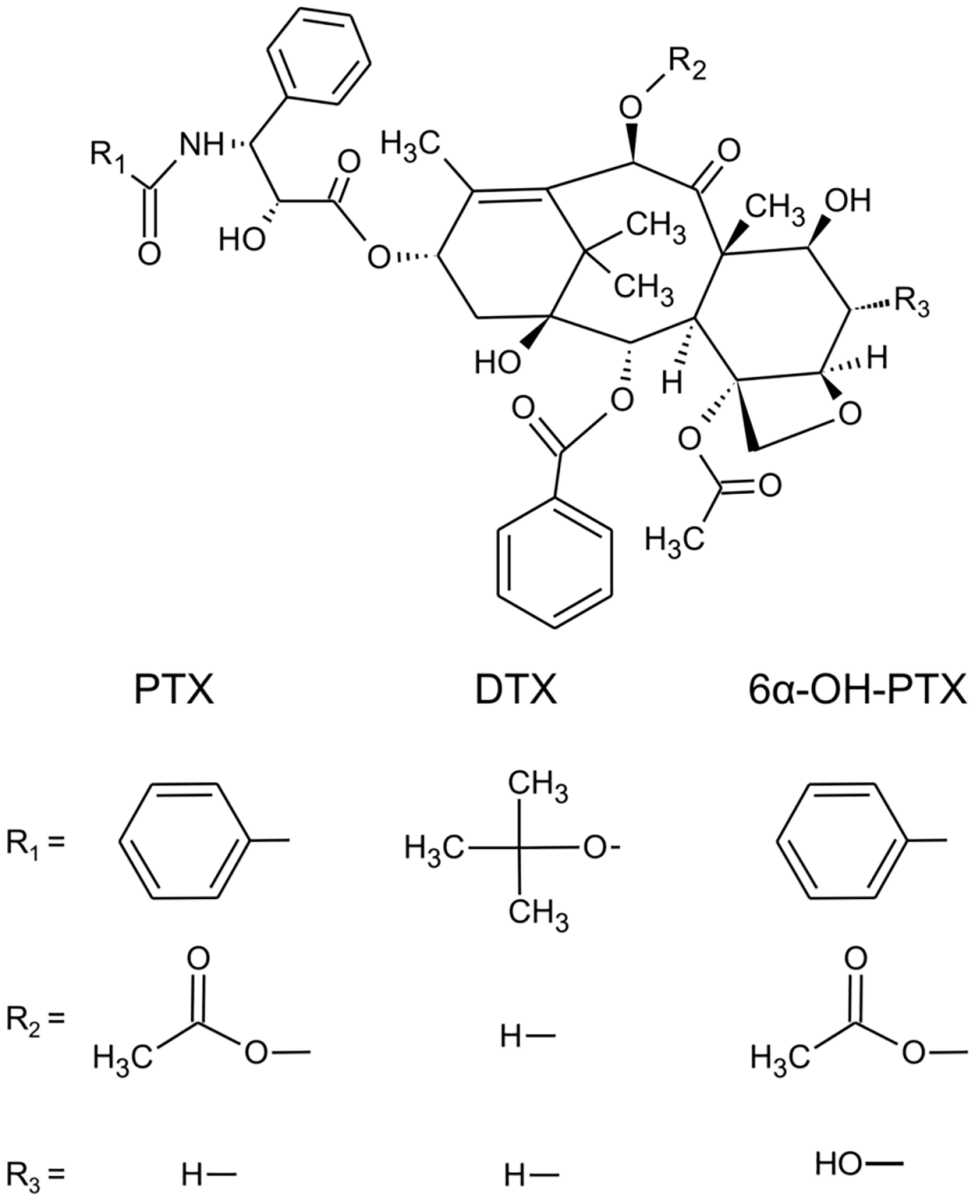
Chemical structures of PTX, 6α-OH-PTX, and DTX (docetaxel) used as IS.

Paclitaxel (PTX) (Fig 1) is a natural product isolated in the early 1970s from the bark of the Pacific Yew (*Taxus brevifolia*) and approved by the US Food and Drug Administration (FDA) in 1992. PTX has a wide clinical spectrum of activity being used for the treatment of breast-, lung-, head and neck-, and ovarian cancer [1–4]. Less common cancers, such as endometrial, unknown primary, testes, esophageal and Kaposi’s sarcoma, also have meaningful response rates to PTX either alone or in combination with other agents [5–7]. High inter- and intra-patient variability in PTX pharmacokinetics and the relationship between haematological toxicity and plasma exposure make Therapeutic Drug Monitoring (TDM) necessary in order to treat patients with the correct dose [8]. In fact, the longer the period that PTX plasma concentration is over 0.05 μM (about 43 ng/mL), the higher the risk for severe neutropenia [8]. Moreover, PTX is used in a wide range of doses (80–225 mg/m^2^) and it showed a disproportionate increase in plasma C_max_ (maximum concentration) and AUC (area under the plasma concentration-vs time curve) as the dose increased, suggesting saturation of elimination at higher concentrations of the drug [9,10]. For these reasons TDM, offering the possibility to individually adjust the dose of drugs, could improve the care of patient treated with PTX leading to a personalized therapy.

The aim of this work was to develop and validate a method to perform TDM of PTX, in patients’ plasma, quantifying even its main metabolite, 6α-hydroxy-paclitaxel (6α-OH-PTX). In fact, 6α-OH-PTX retained bone marrow toxicity when tested on human bone marrow cells [11] and Kang et al. demonstrated that PTX cytotoxicity in HL60 and K562 human leukemia cells had been increased in the presence of noncytotoxic concentrations of 6α-OH-PTX [12].

As reported in Table 1, several methods have been published for the determination of PTX in human plasma [13–27]. Anyway, to our knowledge, just seven offer the possibility to quantify even the main metabolite, 6α-OH-PTX.

**Table 1:** List of publications related to LC-MS/MS methods for the quantification of PTX in human plasma samples.

Ref.	Analyte(s)	Plasma volume (μL)	Sample prep.	LLOQ (ng/mL)	ULOQ (ng/mL)
[13]	PTX	500	PP/ (a)SPE	5	500
[14]	PTX	100	(sa)LLE	1	1000
[15]	PTX, 6α-OH-PTX, p-3’-OH-PTX	400	LLE	0.1, 0.1, 0.1	100
[16]	PTX	500	LLE	10	1000
[17]	PTX	200	LLE	0.25	1000
[18]	DTX, PTX, 6α-OH-PTX, p-3’-OH-PTX	250	LLE	2	1000
[19]	PTX	200	LLE	1	1000
[20]	PTX	100	LLE	2	2500
[21]	PTX, 6α-OH-PTX, p-3’-OH-PTX	500	SPE	0.5	7500, 750, 400
[22]	PTX, 6α-OH-PTX, p-3’-OH-PTX	200	LLE	0.25	1000, 100, 100
[23]	PTX	200	LLE	102.1	20420
[24]	PTX	100	PP/SPE	10	2500
[25]	PTX, 6α-OH-PTX, p-3’-OH-PTX	100	LLE	0.5	500
[26]	PTX, 6α-OH-PTX, p-3’-OH-PTX	90	(o)SPE	5, 0.87, 0.87	5000, 870, 435
[27]	PTX, 6α-OH-PTX, p-3’-OH-PTX	200	LLE	0.125, 0.5, 0.125	100

PTX: paclitaxel; 6α-OH-PTX: 6α-hydroxy-paclitaxel; p-3’-OH-PTX: p-3’-hydroxy-paclitaxel; DTX: docetaxel; PP: protein precipitation; SPE: solid-phase extraction; LLE: liquid-liquid extraction. (o): on-line; (a): automatic; (sa): semi-automatic.

Among the seven methods quantifying even 6α-OH-PTX [15,18,21,22,25,26,27], five require a high volume of plasma being the sample size range between 200 and 500 μL [15,18,21,22,27].

Moreover, the extraction method used in these seven published methods, was LLE (liquid-liquid extraction) [15,18,22,25,27] or SPE (solid-phase extraction) [21,26], which are both time-consuming procedures and therefore not suitable for TDM assays.

The last point to note concerns the linearity range of these methods. The lower limit of quantification (LLOQ) of most of the methods appears fitting with the clinical dose ranges. However, the upper limit of quantification (ULOQ) is not appropriate to quantify PTX and 6α-OH-PTX in samples collected in a dose-escalation study. Moreover, in the literature, the reported range of median (25th-75th percentile) C_max_ is 2,65–8,20 μg/mL following a dose range of 135–240 mg/m^2^ [28]. In fact, five methods are characterized by a PTX ULOQ equal or less than 2500 ng/mL [15,18,22,25,27]. The two methods with the highest ULOQs (5000 ng/mL [26] and 7500 ng/mL [21]) are instead marked by other limitations, such as an unsatisfactory LLOQ (5 ng/mL) for PTX [26] or a very large (500 μL) plasma volume requirement [21].

Therefore, in order to quantify PTX and 6α-OH-PTX in human plasma samples we developed and validated a high-performance liquid chromatography-tandem mass spectrometry (HPLC-MS/MS) method. This method resulted sensitive, specific, and rapid requiring a small volume of plasma sample (100 μL) and only a simple protein precipitation as extraction process.

## Experimental

### Standards and chemicals

Analytical reference standards of PTX (2α,4α,5β,7β,10β,13α-4,10-Bis(acetyloxy)-13-{[(2R,3S)-3-(benzoylamino)-2-hydroxy-3-phenylpropanoyl]oxy}-1,7-dihydroxy-9-oxo-5,20-epoxytax-11-en-2-yl benzoate, batch 061M1664V, purity ≥97%), and docetaxel (DTX, 1,7β,10β-trihydroxy-9-oxo-5β,20-epoxytax-11-ene-2α,4,13α-triyl 4-acetate 2-benzoate 13-{(2R,3S)-3-[(tert-butoxycarbonyl)amino]-2-hydroxy-3-phenylpropanoate}, batch 1425738V, purity ≥97%), used as Internal Standard (IS), were purchased from Sigma-Aldrich Co. (Milan, Italy). 6α-OH-PTX (batch 1JAB113-2, purity ≥98%) was purchased from Toronto Research Chemicals, Inc. (North York, Ontario, Canada). LC-MS grade acetonitrile and formic acid were purchased from Sigma-Aldrich Co. Filtered; LC-MS grade methanol was purchased from VWR (Radnor, Pennsylvania, USA); deionized water was obtained from a Milli-Q Plus system (Millipore, Billerica, MA, USA). Control human plasma/K2-EDTA, used to prepare daily standard calibration curves and quality control (QC) samples was provided by the transfusion unit of the National Cancer Institute (Aviano, Italy) from healthy volunteers.

### Standards and quality control solutions

To prepare standards and quality controls solutions, two different stock solutions for PTX and 6α-OH-PTX were prepared in methanol at the concentration of 2000.0 μg/mL for PTX and 100.0 μg/mL for 6α-OH-PTX. For the IS, the stock solution was prepared in methanol at the concentration of 100 μg/mL. All these solutions have been frozen and stored at -80°C. In order to obtain the working solutions to be used to prepare the plasma standard points of the calibration curve (from G to A) and the plasma QC samples (L-low, M-medium, and H-high), the stock solutions of PTX and 6α-OH-PTX were mixed and diluted with methanol. The final concentrations obtained were: 0.02, 0.20, 1.00, 5.00, 20.00, 100.00, 200.00 μg/mL (from G to A) and 0.06, 12.50, 150.00 μg/mL (QCL, M, and H) for PTX, and 0.02, 0.10, 0.50, 1.00, 2.00, 10.00, 20.00 μg/mL (from G to A) and 0.06, 1.50, 15.00 μg/mL (QCL, M, and H) for 6α-OH-PTX. The working solution of IS was prepared at the concentration of 4 μg/mL by diluting, with methanol, the stock solution. Several aliquots of these solutions were kept in polypropylene tubes at -80°C.

### Preparation of standards and quality control samples

The calibration curves were prepared freshly every day during the validation study and were made up of seven standard points. To prepare the plasma standard points and QCs samples, 5 μL of the respective working solution (see section above) were added to 95 μL of pooled blank human plasma to obtain the final concentration. The achieved concentrations are shown below: 1.00, 10.00, 50.00, 250.00, 1000.00, 5000.00, 10000.00 (G to A) and 3.00, 625.00, 7500.00 (QCL, M, H) ng/mL for PTX, and 1.00, 5.00, 25.00, 50.00, 100.00, 500.00, 1000.00 (G to A) and 3.00, 75.00, 750.00 (QCL, M, H) ng/mL for 6α-OH-PTX. Each calibration curve was prepared including a blank and a zero blank, which are plasma samples processed without and with IS, respectively. Three QC samples for each concentration level (L, M, and H) were used for every analyses. Immediately after the preparation of 6 mL-solution of each QC, (adding 300 μL of each working QC solution to 5.7 mL of control human plasma) several aliquots (100 μL) of the three QCs were stored at −80°C. These aliquots have been used to check PTX and 6α-OH-PTX stabilities and as controls for future assays. The calibration curve and QCs samples were processed as described in the following paragraph.

### Processing samples

The plasma samples were thawed at room temperature, vortexed for 10 s and centrifuged, for 10 min, at 3000 g and at nominally 4°C. Then, 100 μL of standard, QC and actual plasma samples were inserted into a suitable polypropylene tube and added by 5 μL of the IS working solution (4 μg/mL). The mixture, thus obtained, was then vortexed for 10 s. Afterwards, 400 μL of CH_3_OH with 0.1% of HCOOH were added to obtain the protein precipitation. Each sample was vortexed for 10 s and centrifuged, for 15 min, at 16000 g and at nominally 4°C. After that, 150 μL of the obtained supernatant were directly inserted into an autosampler glass vial for the LC-MS/MS analysis. In order to minimize the possible carry-over effect, three mobile phases and one blank plasma sample were run after the ULOQ to guarantee that no peak higher than 10% of LLOQ was detected. With this aim, during the quantification of patients’ samples, they were analysed on the basis of pharmacokinetic evaluations (from the lowest to the highest concentration), and three mobile phases were run between successive test samples.

### Chromatographic conditions

The HPLC system consisted of a SIL-20AC XR auto-sampler and LC-20AD UFLC XR pumps (Shimadzu, Tokyo, Japan). The chromatographic separation of the samples were conducted on a SunFire^™^ C18 column (3.5 μM, 92 Å, 2,1 x 150 mm) coupled with a Security Guard Cartridge (SunFire^™^ C18 2.1 x 10 mm), both provided by Waters (Milford, MA, USA) and thermostatically controlled at 30°C. The mobile phases (MP) were 0.1% HCOOH/bidistilled water (MPA) and 0.1% HCOOH/CH_3_CN (MPB). In the presented method, the following linear gradient was used, with a flow rate of 0.2 mL/min: (step 1) from the initial condition of 60% MPA to 0% over 12 min; (step 2) kept constant for 2 min; (step 3) from 0% MPA to the initial condition over 1 min; (step 4) reconditioning for 6 min. The total run time was 21 min.

### Mass spectrometry

The HPLC system was coupled with an API 4000 triple quadrupole mass spectrometer AB SCIEX (Massachusetts, USA). The optimization of the MS parameters was conducted using standard solutions of each analyte prepared in 0.1% HCOOH acetonitrile/water (1:1) at the concentration of 50 ng/mL and infused at a flow rate of 10 μL/min. The mass spectrometer worked in positive ion mode and was equipped with a TurboIonSpray source operating at 250°C and with the ion spray voltage set to 5500 V. Zero air was employed as nebulizer gas (50 psi) and as heater gas (50 psi) while nitrogen as curtain gas (20 psi) and collision gas (CAD), (at medium intensity). Data were processed with Analyst 1.5.2 and the quantification of the peaks was done with MultiQuant 2.1 (software package AB SCIEX).

### Validation Study

The validation study of the proposed method was conducted as already reported in our previously published work related to a LC-MS/MS method for the simultaneous quantification of irinotecan and its main metabolites in human plasma [29], i.e. as required by the European Medicines Agency (EMA) and the FDA guidance on bio-analytical method validation [30–32]. Thus, the method was validated and the evaluated parameters are the following: recovery, linearity, intra- and inter-day precision and accuracy, limit of detection (LOD), lower limit of quantification (LLOQ), selectivity, matrix effect, stability and reproducibility.

#### Recovery

The percentage of PTX and 6α-OH-PTX extraction recovery was determined at the three QC plasma concentrations (L, M, and H) and at 200 ng/mL for IS. The extraction recovery was evaluated in quintuplicate for each compound. The peak areas of PTX and 6α-OH-PTX, extracted from plasma QC samples, were compared to those from external standards prepared in methanol. The same comparison was performed to determine the recovery of IS. Moreover, for each analyte and IS, the percentage recovery was even determined, in five replicates, by comparing the peak area of the analytes extracted from plasma with the peak area of the extracted matrix added with the same amount of the analytes or IS.

#### Linearity

To validate the linearity, calibration curves were prepared as described in the section “Preparation of standard and quality control samples” over five different working days. The LC-MS/MS peak-area ratios of each analyte/IS compared to the nominal concentrations of each standard point were plotted using a least-squares linear regression. Moreover, to generate the calibration curves a weighted quadratic regression function (1/x^2^) was applied. The linearity of the standard curves was checked by calculating the Pearson’s determination coefficient R^2^ and by comparison of the true and back-calculated concentrations of the calibration standards. The accuracy of back-calculated concentration values of each point had to be within 85–115% of the theoretical concentration and within 80–120% at the LLOQ. Moreover, a minimum of six out of seven standards had to meet these criteria, including the LLOQ and the highest calibrator, ULOQ.

#### Intra- and inter-day precision and accuracy

The precision and accuracy of the presented method were evaluated by analysing three replicates of QC samples (the nominal concentrations of QCL, -M, and—H are reported in the section “Preparation of standards and quality control samples”) within a single-run analysis for intra-day assessment and over five different working days for inter-day assessment. In this latter case, standard calibration curves were freshly prepared and plotted on each of the five days of the validation study. The method precision, at each concentration, was reported as the coefficient of variation (CV%). CV% was defined expressing the standard deviation as a percentage of the mean calculated concentration. The accuracy of the method was determined by expressing the mean calculated concentration as a percentage of the nominal concentration. The measured concentration for at least six out of nine QC samples had to be within 15% of the nominal value, in each run. Moreover, only one QC sample, at each concentration level, could be excluded.

#### Limit of detection, limit of quantification, selectivity and matrix effect

The LOD is the concentration at which the signal-to-noise ratio (S/N) is at least 3. The LLOQ, the concentration of the lowest standard (G), was defined as the lowest concentration that could be measured with a precision within 20% and accuracy between 80% and 120%. Moreover, at the LLOQ the S/N ratio should be at least 5. The LLOQ of the present method was assessed as reported in the section “Preparation of standards and quality control samples”: by adding G working solution to six samples of blank human plasma to obtain the final concentrations. Selectivity was proved using six independent sources of blank human plasma spiked with the analytes at the LLOQ and individually analysed and evaluated for interferences. As already described for LLOQ, also selectivity had to have acceptable accuracy (≤20%) and precision (between 80% and 120%).

During the validation study, matrix effects on the quantification of the two analytes, PTX and 6α-OH-PTX, were also tested. These phenomena arise due to effects of plasma matrix endogenous components on the ionization of the analytes to quantify and IS. Matrix effects were investigated on six independent sources of blank human plasma. They were evaluated by calculating the ratio of the peak area in presence of matrix to the peak area in absence of matrix at the three QC concentrations (L, M, and H) of PTX and 6α-OH-PTX. The CV should be within 15%. Moreover, during the development of the chromatographic method, these phenomena related to plasma matrix endogenous components were investigated by means of the post-column infusion: a constant flow of standard solutions of PTX, 6α-OH-PTX and IS, each prepared in 0.1% HCOOH acetonitrile/water 1:1 (50 ng/mL), were infused by a syringe pump during the chromatographic run of an extracted pooled blank human plasma sample. The extracted plasma sample eluted from the LC column and the flow from the infusion pump were combined by means of a zero-dead-volume ‘T’ union and inserted into the mass spectrometer source. A variation in the signal response of the infused analyte, caused by the coeluted interfering compounds, indicates ionization enhancement or suppression.

#### Stability

The stability of PTX and 6α-OH-PTX was assessed by analysing QC samples (at the three concentrations L, M, and H) during sample storage and handling procedures. Bench-top stability of the analytes in plasma matrix was determined after 4 h at room temperature. The stability of the processed QC samples was assessed in the autosampler by repeatedly analysing the extracts 24, 48 and 72 h after the first injection. Moreover, a freshly prepared aliquot of each QC sample concentration was processed and analysed, and then again after one and two freeze/thaw cycles in order to check freeze/thaw stability. Long-term stability was assessed in plasma as well as in working solutions (methanol matrix) stored at approximately −80°C. PTX and 6α-OH-PTX were considered stable when the differences between the freshly prepared samples and the testing samples did not exceed 15% from the nominal concentrations at each QC concentration (L, M, and H).

#### Reproducibility

The evaluation of bioanalytical methods by re-analysis of incurred samples should be performed as an additional measure of assay reproducibility [33], as indicated in the revised version of the FDA Guidance for Industry on Bioanalytical Method Validation [31]. Incurred Sample Reanalysis (ISR) is a very important component of the bioanalytical method validation. In fact, ISR is intended to verify the reliability of the reported analyte concentrations and it is conducted by repeating the analysis, of a subset of subject samples in separate runs, on different days, with the same bioanalytical method procedures. Therefore, the reproducibility of the present method was assessed by re-analysing the incurred samples of one patient, enrolled in a clinical study, in a further analytical session. The study samples for this reanalysis were chosen in order to guarantee an adequate coverage of the pharmacokinetic profile. To do that, in the re-analysis, samples at high concentration (around the C_max_) and at low concentration (samples representative of the elimination phase) were included. The two analyses can be considered equivalent if the 67% of the results percentage difference [(repeat-original)*100/mean] is within 20% [31].

### Application of the method to clinical samples

The proposed method was applied to a phase Ib clinical trial of weekly PTX in ovarian cancer patients in order to obtain the pharmacokinetics of PTX and its principal metabolite. In this dose-escalation study, PTX was administered weekly as 60 min intravenous infusion on a four-week treatment cycle. For the pharmacokinetic analysis, serial blood samples were collected into tubes containing K2-EDTA (as the anticoagulant) at the following time-points during the first chemotherapy cycle: before drug administration, and at 1.0, 1.25, 1.50, 2.0, 4.0, 8.0, 25.0, 49.0 h following the start of the PTX infusion. By centrifugation of the blood samples at 3000 g for 10 min at 4°C, plasma was obtained immediately after the collection. Afterwards, the plasma was separated, split into 2 suitable tubes and stored, at -80°C, as two independent aliquots. The principal pharmacokinetic parameters were calculated using WinNonlin software with non-compartmental analysis.

#### Ethics statement regarding human samples

The phase Ib study entitled “A genotype-guided phase I study for weekly paclitaxel in ovarian cancer patients” was approved by the ethics committee of the National Cancer Institute of Aviano (CRO, Italy) and by Istituto Superiore di Sanità (ISS, Rome, Italy), EudraCT number: 2010-021619-18. The study was conducted according to the principles expressed in the Declaration of Helsinki. All blood samples were collected only after the signature of the informed consent from all the patients enrolled in the clinical study.

## Results and discussion

### HPLC-MS/MS method

**Fig 2:**
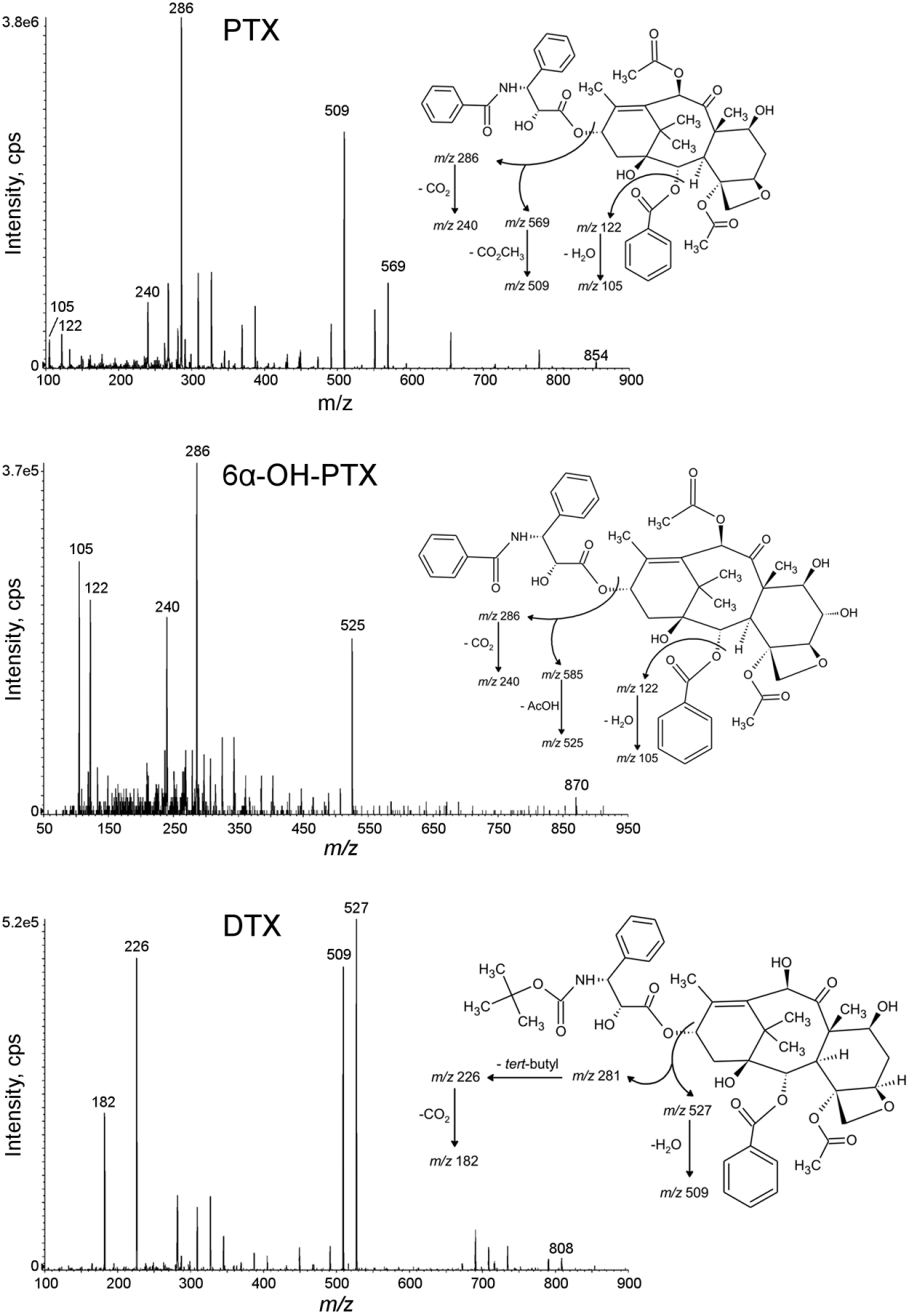
MS/MS mass spectra of PTX and 6α-OH-PTX with chemical structures and identification of the main fragment ions.

**Fig 3:**
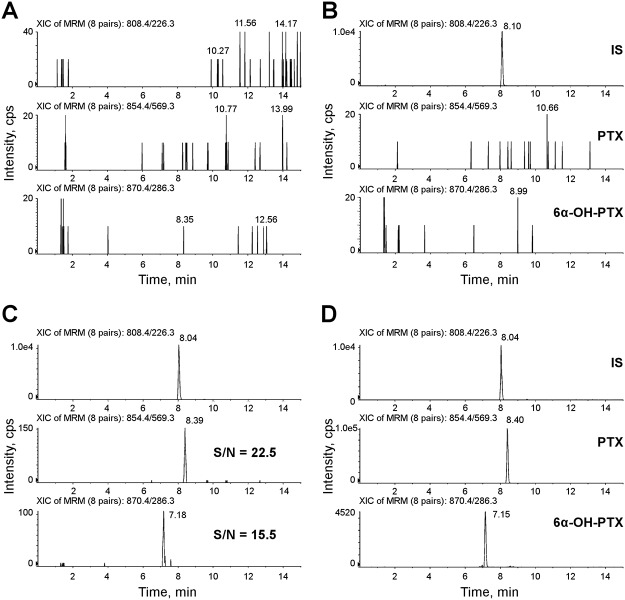
Representative SRM chromatograms. (A): SRM chromatograms of a human blank plasma sample; (B): SRM chromatograms of a human blank plasma sample with IS added; (C): S/N of PTX and 6α-OH-PTX at the LLOQ (1.00 ng/mL for both analytes); (D): SRM chromatograms of an extracted plasma sample of a treated patient showing IS, PTX (1997.39 ng/mL) and 6α-OH-PTX (93.67 ng/mL).

An infusion of PTX, 6α-OH-PTX and IS was used to optimize the conditions of mass spectrometer. Working in positive ion mode, PTX and 6α-OH-PTX formed mainly a protonated molecule [M+H]^+^. In Table 2, the mass spectrometer parameters and ion transitions of PTX and 6α-OH-PTX are reported. Fig 2 represents the fragmentation patterns of each compound and the daughter ion with the highest signal was chosen as quantifier: 854.5 >569.3 *m/z* for PTX, 870.5>286.3 *m/z* for 6α-OH-PTX, and 808.5>226.3 *m/z* for IS. Typical SRM chromatograms are reported in Fig 3: an extracted blank plasma sample (A); an extracted blank plasma sample added with IS (B); an extracted plasma sample at the LLOQ added with IS (C), and an extracted plasma sample of a patient (D). This last sample was drawn at the end of the intravenous infusion (1-h, 80 mg/m^2^ of PTX) and the peaks correspond to concentrations of 1997.39 ng/mL of PTX and 93.67 ng/mL of 6α-OH-PTX. The elution resulted rapid and selective, being the peaks separated within 10 min: PTX, 6α-OH-PTX and IS were eluted at approximately 8.40, 7.15 and 8.04 min, respectively, and, at these retention times, no interfering peaks were observed. Moreover, the analysis of six independent sources of blank human plasma confirmed the specificity of the method.

**Table 2:** Source- and compound-dependent parameters and ion transitions of each analyte and IS used for the mass spectrometer method.

Precursor Ion				Daughter ion		
Analyte	Q1 (amu)	DP (volts)	EP (volts)	Q3 (amu)	CE (volts)	CXP (volts)
PTX	854.5	63	9	569.3	15	18
				286.3	23	7
				105.1	95	19
6α-OH-PTX	870.5	63	8	286.3	23	7
				105.1	94	18
				525.3	22	16
DTX	808.5	50	7	226.3	23	22
				527.3	14	16

The dwell time of each transition was set up at 50 msec. DP: declustering potential; EP: entrance potential; CE: collision energy; CXP: collision cell exit potential.

### Validation of the method

#### Recovery

The proposed method is based on a simple protein precipitation of the plasma samples with four volumes of 0.1% HCOOH/CH_3_OH. The recovery resulted in the range 92.4–95.7% (CV ≤6.9%) for PTX and 93.4–97.7% (CV ≤5.5%) for 6α-OH-PTX, as shown in Table 3, while the recovery of IS was 101.2% (CV 4.4%).

**Table 3:** Recovery of the analytes and the IS from human plasma.

Analyte	Nominal concentration (ng/mL)	Recovery (%) ± SD	CV %
PTX	3	95.7 ± 6.4	6.6
	625	92.4 ± 6.4	6.9
	7500	93.8 ± 1.3	1.4
6α-OH-PTX	3	94.2 ± 1.9	2.0
	75	97.7 ± 5.4	5.5
	750	93.4 ± 2.6	2.8
DTX (IS)	200	101.2 ± 4.5	4.4

#### Linearity

The accuracy and precision for each calibration points of PTX and 6α-OH-PTX, freshly prepared every day during the validation study, are reported in Table 4. The standard curves were prepared on five different days and showed good linearity, being the Pearson’s coefficient of determination R^2^ ≥0.9948 for each run. Acceptable results were obtained within the validated range of 1.00–10000.00 ng/mL for PTX and of 1.00–1000.00 ng/mL for 6α-OH-PTX: the mean accuracy resulted in the range 89.8–107.6% for PTX and 87.0–106.8% for 6α-OH-PTX and the precision, expressed as CV%, ranged from 0.6% for the LLOQ to 7.9% for PTX and from 0.8 to 7.1% for 6α-OH-PTX.

**Table 4:** Linearity, accuracy and precision data for calibration curves of PTX and its metabolite 6α-OH-PTX.

**PTX**			
**Nominal conc. (ng/mL)**	**Mean ± SD**	**Precision %**	**Accuracy %**
1	0.99 ± 0.01	0.6	99.4
10	10.59 ± 0.68	6.4	105.9
50	50.91 ± 1.74	3.4	101.8
250	269.00 ± 9.05	3.4	107.6
1000	1020.79 ± 52.28	5.1	102.1
5000	4488.84 ± 161.87	3.6	89.8
10000	9292.7 ± 736.14	7.9	92.9
**6α-OH-PTX**			
**Nominal conc. (ng/mL)**	**Mean ± SD**	**Precision %**	**Accuracy %**
1	1.01 ± 0.01	0.8	100.8
5	4.82 ± 0.27	5.7	96.5
25	21.74 ± 0.48	2.2	87.0
50	53.40 ± 3.63	6.8	106.8
100	102.43 ± 4.12	4.0	102.4
500	512.77 ± 17.90	3.5	102.6
1000	1011.36 ± 71.36	7.1	101.1

#### Intra- and inter-day precision and accuracy

The intra- and inter-day accuracy and precision (CV%) obtained are reported in Table 5. The precision of the proposed method was confirmed by the intra- and inter-day CV ≤9.2% and ≤7.0% for PTX and ≤7.9% and ≤9.9% for 6α-OH-PTX. Moreover, the intra- and inter-day accuracy were within the range 91.1–98.4% and 94.0–104.8% for PTX and 92.8–103.3% and 99.5–104.0% for 6α-OH-PTX.

**Table 5:** Intra and inter-day precision and accuracy of the method for the analysis of PTX and its metabolite 6α-OH-PTX in human plasma samples.

**Intra-day (N = 5)**				
**Analytes**	**Nominal concentration (ng/mL)**	**Mean ± SD**	**Precision %**	**Accuracy %**
PTX	3	3.18 ± 0.29	9.2	94.4
	625	685.97 ± 40.72	5.9	91.1
	7500	7619.62 ± 370.09	4.9	98.4
6α-OH-PTX	3	2.91 ± 0.23	7.9	103.3
	75	80.85 ± 4.24	5.2	92.8
	750	808.02 ± 50.47	6.2	92.8
**Inter-day (N = 15)**				
**Analytes**	**Nominal concentration (ng/mL)**	**Mean ± SD**	**Precision %**	**Accuracy %**
PTX	3	3.14 ± 0.19	5.9	104.8
	625	644.20 ± 45.22	7.0	103.1
	7500	7047.92 ± 477.06	6.8	94.0
6α-OH-PTX	3	2.99 ± 0.29	9.9	99.5
	75	77.99 ± 5.73	7.3	104.0
	750	766.42 ± 58.58	7.6	102.2

#### Limit of detection, limit of quantification, selectivity and matrix effect

The LOD resulted 0.13 ng/mL for PTX and 0.19 ng/mL for 6α-OH-PTX. As shown in panel C of Fig 3, reporting the S/N values obtained (22.5 for PTX and 15.5 for 6α-OH-PTX), the LLOQ was fixed at 1 ng/mL for both PTX and 6α-OH-PTX. The accuracy and CV% were, respectively, 109.8% and 5.0% for PTX and 106.5% and 8.1% for 6α-OH-PTX.

The method resulted not affected by endogenous components in the matrix or other components in the sample: by spiking six different sources of human plasma with the analytes at the LLOQ concentration, the accuracy and CV% were, respectively, 107.5 and 12.5% for PTX and 104.0% and 6.5% for 6α-OH-PTX. No matrix effect of ion suppression or enhancement was observed because no significant variations (<15%) in the peak area of each analyte in the six lots of matrices were detected.

#### Stability

Both PTX and 6α-OH-PTX resulted stable in human plasma for 4 h at room temperature and, after extraction, for 72 h in the autosampler (4°C) (S1 Table). Moreover, the two compounds resulted stable in human plasma over two freeze/thaw cycles: CV% and accuracy were ≤3.1% and within 102.4–113.1% for PTX, and ≤12.8% and within 94.0–112.2% for 6α-OH-PTX (S2 Table). The long-term stability in human plasma was assessed after 7 months of storage at approximately -80°C: CV% and accuracy obtained were, respectively, ≤4.5% and within 94.6–103.2% for PTX, and ≤4.9% and within 89.6–102.8% for 6α-OH-PTX (S2 Table). The stability of the standard working solutions of PTX and 6α-OH-PTX was assessed after 27 months of storage at −80°C: CV% and accuracy were ≤3.6% and within 106.6–113.3% for PTX, and ≤8.8% and within 105.6–113.6% for 6α-OH-PTX (S3 Table).

#### Reproducibility

**Fig 4:**
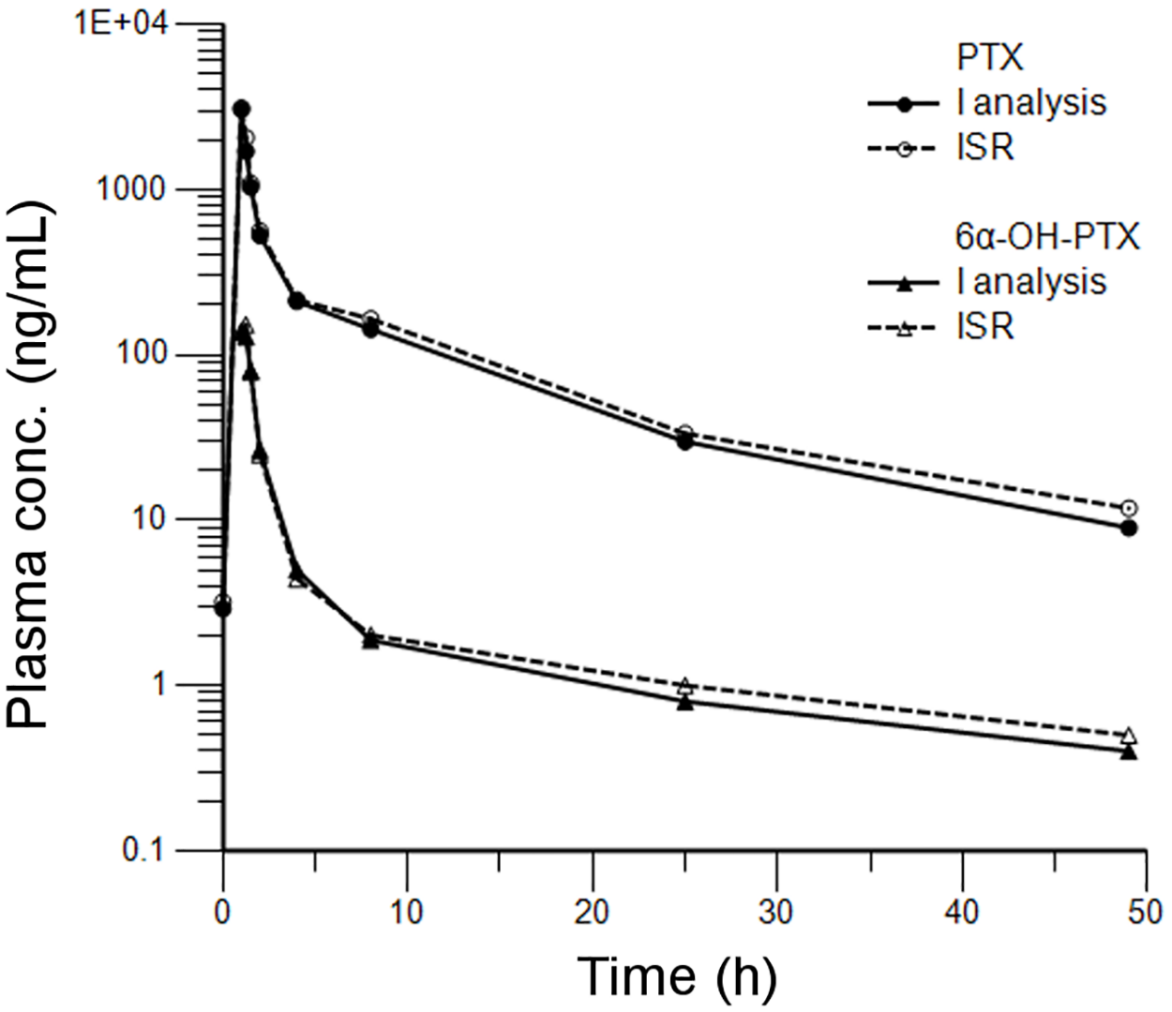
Re-analysis of incurred plasma samples of one patient treated at the dose of 100 mg/m^2^ of PTX during the first chemotherapy cycle.

The re-analysis of plasma samples from one patient (treated at the dose of 100 mg/m^2^) demonstrated a good reproducibility and accuracy of the method. The concentrations of PTX and 6α-OH-PTX, determined during the two different analytical runs, were indeed very similar in all samples: the percentage difference resulted within 20% in ≥94.1 and ≥91.7% for all the re-analysed samples for PTX and 6α-OH-PTX, respectively (Fig 4).

### Pharmacokinetic study

**Fig 5:**
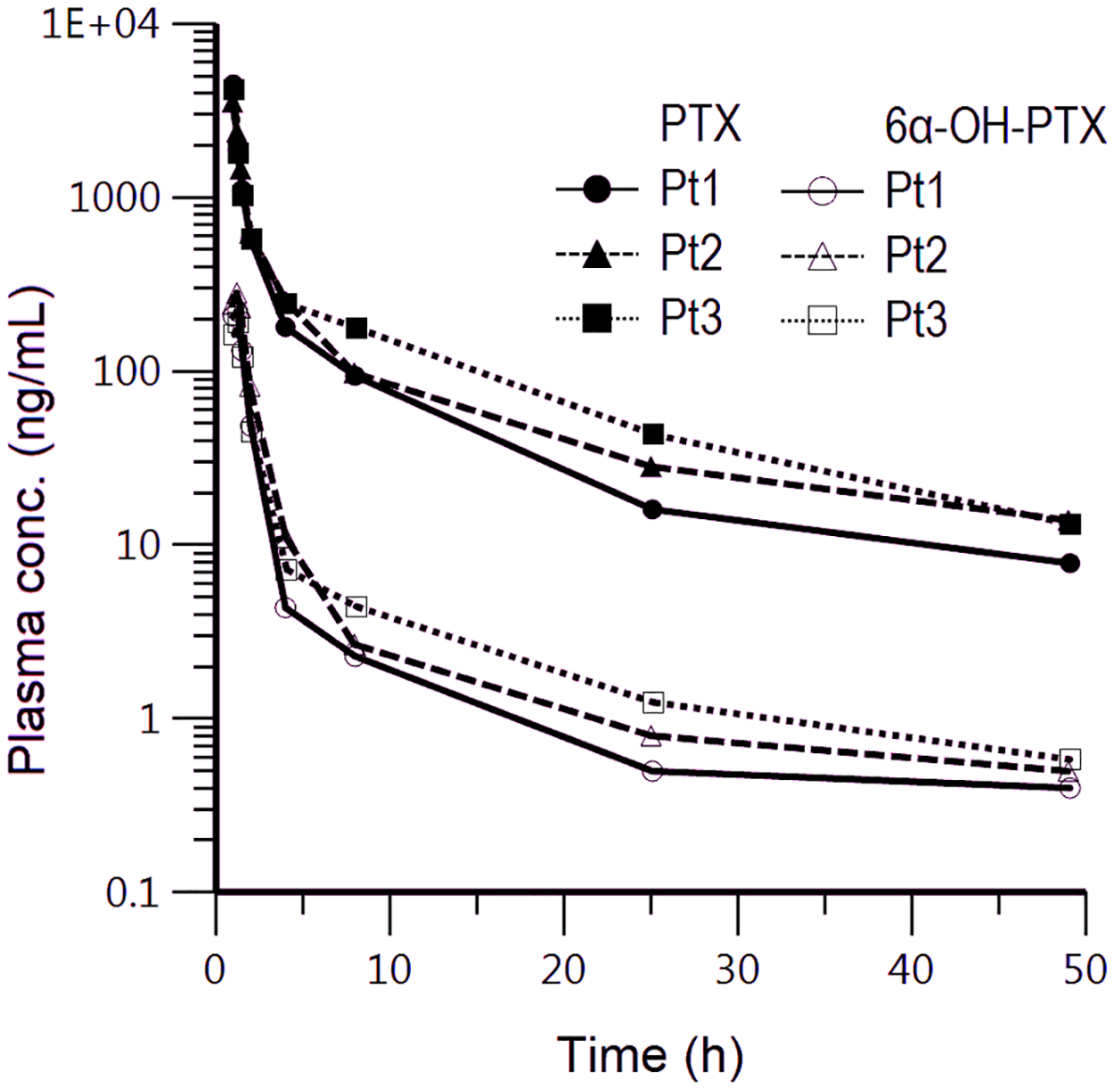
Plasma concentration-*vs*-time profiles of PTX and its main metabolite 6α-OH-PTX in three patients with advanced ovarian cancer. Patient 1, 2, and 3 received 110 mg/m^2^ of PTX as 1-h intravenous infusion during the first chemotherapy cycle.

The present method was successfully applied to the pharmacokinetic study of PTX and 6α-OH-PTX in ovarian cancer patients enrolled in an ongoing genotype-guided phase I study and treated with weekly PTX. In order To quantify patients’ samples, a standard curve was freshly prepared every working day and the concentrations of samples were back-calculated from the standard curve. The highest concentrations found in all the patients’ samples analysed till now were within the dynamic range of the assay. For this reason, it was not necessary any further dilution steps, even if the analysis independence from the dilution was previously assessed at two dilution factors: 1:10 and 1:100. Here we reported the pharmacokinetic data related to three patients (Pt 1, 2, 3) receiving 110 mg/m^2^ of PTX to confirm the method applicability to samples collected from patients. The pharmacokinetic profiles were plotted. Fig 5 shows the plasma concentration versus time curves of PTX and 6α-OH-PTX determined in these patients, during the first cycle of therapy, using the method described before. The profiles were well described within 49 h. The maximum concentrations of PTX and 6α-OH-PTX in patient 1 were observed as 4471.82 and 220.98 ng/mL respectively at 1 h and 1.25 h after the beginning of the infusion. The experimental AUC (AUC_exp_, area under the curve of PTX and 6α-OH-PTX plasma concentration vs. time from 0 to 49 h) were 6345.63 and 336.09 ng/mL*h for PTX and 6α-OH-PTX, respectively. The terminal elimination half-life (t_1/2_) of PTX and 6α-OH-PTX were 11.87 and 7.02 h, respectively. Regarding the patient 2, C_max_ and AUC_exp_ were 3530.57 ng/mL and 6559.601 ng/mL*h for PTX and 282.02 ng/mL and 471.75 ng/mL*h for 6α-OH-PTX, while t_1/2_ of PTX and 6α-OH-PTX were 14.93 and 5.22 h, respectively. Lastly, patient 3 exhibited PTX C_max_ comparable (4169.55 ng/mL) to the other two patients but a quite higher AUC_exp_, being 7974.78 ng/mL*h. The same behaviour was noticed for 6α-OH-PTX. In fact, C_max_ and AUC_exp_ were 190.75 ng/mL and 359.03 ng/mL*h. Patient 3 showed an elimination t_1/2_ of 11.09 and 14.35 h for PTX and 6α-OH-PTX, respectively.

### Discussion

Paclitaxel has been studied at doses of 15–825 mg/m^2^, infused over 0.5 to 96 h and administered both weekly and every 3-weeks dosing cycles. In the literature, the reported range of median (25th-75th percentile) C_max_ is 2,65–8,20 μg/mL following a dose range of 135–240 mg/m^2^ [28]. With the aim of developing a method suitable for both a routine clinical application and a dose-escalation study, we needed a method employing a small volume of plasma, with a very simple extraction method, and characterized by a wide concentration range. In fact, the concentration range should be proper to accurately determine both the C_max_, in samples of patients enrolled in high-dose PTX clinical studies, and the elimination phase of the analytes.

With this purpose, we developed a new HPLC–MS/MS method proper to quantify not only PTX but even its main metabolite. The method resulted characterized by high selectivity and sensitivity further guaranteed by working in the Selected Reaction Monitoring (SRM) acquisition mode [34]. Furthermore, with the aim to quantify both PTX and 6α-OH-PTX on plasma collected by pluri-treated patients, three transitions for each analytes (one transition chosen as quantifier and the other two as qualifiers) were followed to detect possible interferences. We investigated a range of concentrations (1–10000 ng/mL for PTX and 1–1000 ng/mL for 6α-OH-PTX) that we expected to cover those found in the patients’ plasma. This method requires just 100 μL of plasma sample and a simple treatment with 0.1% formic acid in methanol as extraction procedure from the biological matrix. Moreover, to assure the robustness and reliability of our method, we validated it according to the FDA-EMA guidances on bioanalytical method validation [30–32]. The developed and validated method was swimmingly applied to a pharmacokinetic study of weekly PTX administered in ovarian cancer patients included in a genotype-guided phase I study, that is a dose-increment study. The main aim of this phase I trial was the definition of the maximally-tolerated dose of weekly PTX, as first-line therapy, on the basis of the *ABCB1* genotype.

During the validation process we assessed the independence of analysis from the dilution (dilution factors of 1:10 and 1:100). However, the highest concentrations found in patients samples were within the calibration range of the method, thus making this assay suitable to quantify all PTX doses nowadays employed in clinical practice. In addition, as suggested by the FDA guidelines [31], we evaluated the reproducibility of the developed method even by re-analysis of incurred samples.

## Conclusions

PTX is used in a wide range of doses (80–225 mg/m^2^) alone or in multidrug regimens for the therapy of several solid tumours. PTX pharmacokinetics is marked by a high inter- and intra-patient variability having repercussion on both response and toxicity to the treatment. Moreover, a relationship between haematological toxicity and plasma exposure was found. For these reasons, to individually adjust the dose by means of TDM is necessary in order to treat patients with the correct dosage and therefore to optimize clinical outcomes.

The developed and validated bioanalytical method, requiring a simple deproteinization and HPLC–MS/MS determination, could be used for TDM assays of PTX. This method requires just 100 μL of plasma, is rapid, selective, highly sensitive, precise, accurate and able to quantify even PTX main metabolite, 6α-OH-PTX. It was successfully validated according to the FDA-EMA guidances on bioanalytical method validation. The method has been used to measure plasma concentrations of PTX and 6α-OH-PTX and to determine the pharmacokinetics of these analytes in plasma samples of cancer patients recruited in an ongoing phase I study.

## Supporting information

S1 Table Short term stability of PTX and its main metabolite 6α-OH-PTX in human plasma samples. (DOCX)

S2 Table Stability of PTX and 6α-OH-PTX, in human plasma samples, after 2 freeze-thaw cycles and after 7 months of storage at -80°C. (DOCX)

S3 Table Stability of the working solutions of PTX and 6α-OH-PTX stored at -80°C over 27 months. (DOCX)
