# Supplementary material for: A new high-performance liquid chromatography-tandem mass spectrometry method for the determination of paclitaxel and 6α-hydroxy-paclitaxel in human plasma: Development, validation and application in a clinical pharmacokinetic study
Source: PLoS One. 2018 Feb 23;13(2):e0193500. doi: 10.1371/journal.pone.0193500 (PMC5825125; doi:10.1371/journal.pone.0193500)
Supplement: S1 Table — (DOCX) [file pone.0193500.s001.docx]

**S1 Table.** **Short term stability of PTX and its metabolite 6α-OH-PTX in human plasma samples.**

|  |  | **T = 4h (RT)** | | | **T = 72h in autosampler (4°C)** | | |
| --- | --- | --- | --- | --- | --- | --- | --- |
| **Analytes** | **Nominal conc. (ng/mL)** | **Mean ± SD** | **Prec. %** | **Acc. %** | **Mean ± SD** | **Prec. %** | **Acc. %** |
| **PTX** | 3 | 2.71 ± 0.21 | 7.7 | 90.2 | 3.03 ± 0.03 | 1.1 | 101.1 |
|  | 625 | 561.64 ± 26.83 | 4.8 | 89.9 | 617.20 ± 15.39 | 2.5 | 98.8 |
|  | 7500 | 6658.95 ± 378.35 | 5.7 | 88.8 | 6703.78 ± 435.30 | 6.5 | 89.4 |
| **6α-OH-PTX** | 3 | 2.58 ± 0.02 | 0.7 | 86.0 | 2.74 ± 0.19 | 7.1 | 91.4 |
|  | 75 | 66.60 ± 1.64 | 2.5 | 88.8 | 76.88 ± 1.55 | 2.0 | 102.5 |
|  | 750 | 666.35 ± 36.97 | 5.5 | 88.8 | 725.84 ±25.13 | 3.5 | 96.8 |
