# Supplementary material for: A new high-performance liquid chromatography-tandem mass spectrometry method for the determination of paclitaxel and 6α-hydroxy-paclitaxel in human plasma: Development, validation and application in a clinical pharmacokinetic study
Source: PLoS One. 2018 Feb 23;13(2):e0193500. doi: 10.1371/journal.pone.0193500 (PMC5825125; doi:10.1371/journal.pone.0193500)
Supplement: S2 Table — (DOCX) [file pone.0193500.s002.docx]

**S2 Table.** **Stability of PTX and 6α-OH-PTX, in human plasma samples, after 2 freeze-thaw cycles and after 7 months of storage at -80°C.**

|  |  | **After 2 freeze-thaw cycles** | | | **Stored at -80ºC over 7 months** | | |
| --- | --- | --- | --- | --- | --- | --- | --- |
| **Analytes** | **Nominal conc. (ng/mL)** | **Mean ± SD** | **Prec. %** | **Acc. %** | **Mean ± SD** | **Prec. %** | **Acc. %** |
| **PTX** | 3 | 3.21 ± 0.03 | 0.9 | 107.1 | 2.97 ± 0.11 | 3.8 | 99.0 |
|  | 625 | 706.72 ± 15.66 | 2.2 | 113.1 | 591.47 ± 8.31 | 1.4 | 94.6 |
|  | 7500 | 7683.00 ± 236.01 | 3.1 | 102.4 | 7739.55 ± 354.80 | 4.5 | 103.2 |
| **6α-OH-PTX** | 3 | 2.82 ± 0.36 | 12.8 | 94.0 | 2.69 ±0.1 | 3.8 | 89.6 |
|  | 75 | 84.19 ± 2.57 | 3.1 | 112.2 | 76.19 ± 2.35 | 3.1 | 101.6 |
|  | 750 | 814.86 ± 2.24 | 0.3 | 108.6 | 771.01 ± 37.96 | 4.9 | 102.8 |
