# Supplementary material for: A new high-performance liquid chromatography-tandem mass spectrometry method for the determination of paclitaxel and 6α-hydroxy-paclitaxel in human plasma: Development, validation and application in a clinical pharmacokinetic study
Source: PLoS One. 2018 Feb 23;13(2):e0193500. doi: 10.1371/journal.pone.0193500 (PMC5825125; doi:10.1371/journal.pone.0193500)
Supplement: S3 Table — (DOCX) [file pone.0193500.s003.docx]

**S3 Table.** **Stability of the working solutions of PTX and 6α-OH-PTX stored at -80ºC over 27 months.**

|  |  | **Stored at -80ºC over 27 months** | | |
| --- | --- | --- | --- | --- |
| **Analytes** | **Nominal conc. (ng/mL)** | **Mean ± SD** | **Prec. %** | **Acc. %** |
| **PTX** | 3 | 3.20 ± 0.12 | 3.6 | 106.6 |
|  | 625 | 704.35 ± 4.68 | 0.7 | 112.7 |
|  | 7500 | 8499.14 ± 153.41 | 1.8 | 113.3 |
| **6α-OH-PTX** | 3 | 3.17 ± 0.28 | 8.8 | 105.6 |
|  | 75 | 83.78 ± 0.28 | 0.3 | 111.7 |
|  | 750 | 851.94 ± 4.27 | 0.5 | 113.6 |
